# Effect of training focused on executive functions (attention, inhibition, and working memory) in preschoolers exhibiting ADHD symptoms

**DOI:** 10.3389/fpsyg.2015.01161

**Published:** 2015-08-06

**Authors:** Anna M. Re, Agnese Capodieci, Cesare Cornoldi

**Affiliations:** ^1^Department of Development and Socialization Psychology, University of PadovaPadova, Italy; ^2^Department of General Psychology, University of PadovaPadova, Italy

**Keywords:** ADHD, preschool children, training, executive function, attention, impulsive behavior, working memory

## Abstract

The development of early intervention strategies for children with symptoms of Attention Deficit Hyperactivity Disorder (ADHD) is important because it provides an opportunity to prevent severe problems in the future. The main purpose of this investigation was to determine the efficacy of a group training for the control of attention, working memory and impulsive behaviors, involving 5-year-old children with ADHD symptoms. Twenty-six children with ADHD symptoms and 26 with typical development were randomly divided in two conditions. Thirteen children in each group were assigned to the training condition and the other to the business as usual condition (normal class activity). Children who participated in the intervention showed an improvement in the tasks measuring their control of attention, impulsive behavior, and working memory. Moreover, children with typical development who attended the training also improved their competencies. The results confirm the importance of an early intervention for preschool-age children with ADHD symptoms.

## Introduction

Although it is well-known that ADHD symptoms are linked to a biological predisposition and are often evident during a child’s first few years, the assessment procedures and the subsequent treatment are usually administered when children are well along in the primary grades and have probably been exposed to negative experiences. As a consequence, their ADHD symptoms could have been emphasized by school, failures, and social exclusion. These considerations can make the disorder more resistant to psychological treatment. It is therefore important to consider children younger than six who show ADHD symptoms helping them with an early intervention, as also suggested by previous research showing the impact of the early presence of ADHD symptoms (Sonuga-Barke et al., 2006) and of early intervention (Young and Amarasinghe, 2010) on subsequent growth.

The present study is focused on the effects of an early intervention on executive functions (EFs). EFs are a set of general-purpose control processes that regulate one’s thoughts and behaviors (Miyake and Friedman, 2012), inside of this set we have different skills and abilities like working memory, capacity to suppress inappropriate responses or behaviors, and to shift between different activities. As it is well known from literature, ADHD children have weaknesses in their EFs like attentional control, working memory, and inhibition. In particular important meta-analyses showed impairment of ADHD children in several EFs (e.g., Reid et al., 2005; Willcutt et al., 2005). For example, the meta-analysis of Martinussen et al. (2005) highlighted an ADHD impairment in working memory that was greater in visuo-spatial working memory than in the verbal one. In particular, some studies have found weaknesses in executive functions also in pre-school children who exhibit symptoms of ADHD (Mariani and Barkley, 1997; Schoemaker et al., 2012; Sinzig et al., 2014).

Sonuga-Barke et al. (2002) examined a large sample of pre-schoolers with ADHD and found specific difficulties in the inhibition capacity, planning, and working memory. More recently Re et al. (2010) compared a group of 23 kindergarten children characterized by the presence of ADHD symptoms, and one group of 23 children matched for gender, age, and socioeconomic status, in a visuo-spatial working memory task which required the selective recall of information. Authors found that children with ADHD symptoms performed more poorly than controls and were affected to a particularly high extent by intrusion errors (i.e., recalling of information initially encoded but that needed to be consequently suppressed during the task). In sum, from literature we can argue that executive functions are already impaired in pre-schoolers with symptoms of ADHD and are crucial in the children’s development as they may offer the basis for the self-regulation requests present in later years (Diamond, 2012), suggesting that they could be the object of an early treatment devoted to reduce the presence of ADHD symptoms.

Despite the potential advantages of an early intervention on young children who exhibit ADHD symptoms, intervention studies on children with ADHD symptoms who are younger than six are few. A first reason of this concerns their identification, as symptoms of ADHD at young ages may be unclear, reflect other problems, or simply be due to maturation variations and delays. A second problem concerns the practical and social limitations that interventions on young children with ADHD may meet.

In general, it seems important to devise intervention projects that support young children with ADHD symptoms by possibly involving not only the children but also their schools and families (Sonuga-Barke et al., 2001; Young and Amarasinghe, 2010; DuPaul and Kern, 2011). A recent meta-analysis (Rajwan et al., 2012) found 29 intervention studies on young children, but they mainly concerned parent training (10), teacher training (3), diets (2), nutritional supplements (1), and acupuncture (1). Only four studies considered a direct psychological intervention for the children. This confirms the need of evidence on interventions directly involving children that is also evident at older ages (Evans et al., 2013).

Interventions, that aim directly to teach children some skills to regulate their behavior, are mainly focused on cognitive-behavioral strategies, either starting from external verbal prompts given from an adult trainer and moving towards an internal self-statement made by child, or using contingence analyses and reinforcement techniques without deeply considering the associated neuropsychological problems.

However, there are a few studies on pre-school children involving intervention on EFs (Bergman Nutley et al., 2011; Röthlisberger et al., 2012). In particular, Re and Cornoldi (2007) conducted a pilot study on 5-year-old children with ADHD symptoms and they found that an intervention on attentive control and working memory improved their executive functions and reduced the presence of ADHD symptoms. These results were substantially replicated by another pilot study carried out with first-graders with symptoms of ADHD (Salvaguardia et al., 2009). However, these two studies were preliminary and could not control for a series of intervening variables and for the possibility of carrying out an intervention in the context of everyday school activities. Thorell et al. (2009) investigated the effects of two different trainings, one specific for working memory and the other one for inhibition. Preschool children received computerized training of either visuo-spatial working memory or inhibition for 5 weeks and were then compared with an active control group that had played commercially available computer games, and a passive control group. The results of the study suggested that working memory training can have significant effects also with preschool children and be more effective than inhibition training. Finally, a study with an intervention program on EFs with ADHD children aged between 4 and 5 years was carried out by Halperin et al. (2013). Children and their parents participated in separate group sessions where they played games designed to enhance inhibitory control, working memory, attention, visuo-spatial abilities, planning, and motor skills. Parents were also encouraged to play these games with their children at least 30–45 min/day. It was found that parents and teachers ratings about severity of ADHD symptoms decreased significantly from the pre to the post test.

In sum, studies on cognitive intervention on children’s EFs are showing good improvements. However, but more evidence is needed on specific programs and condition following a protocol that permits repeatable results (Rapport et al., 2013), and on psycho-educational interventions for young children may be carried out at schools, possibly in groups, and in the context of everyday activities. The preliminary available evidence (DuPaul and Kern, 2011) seems promising and shows the long-term effects on the prevention of associated behavioral disruptive problems (Kern et al., 2007).

The present study intends to examine more systematically the effects of the training of executive functions, in particular attentive control, inhibition and working memory, carried out in the context of school activities with groups of preschoolers, including not only children with ADHD symptoms but also typically developing children (TD children). In fact, schools typically require that interventions are carried out during the everyday activities and potentially interest all children. In this way we had the advantage of testing the efficacy of an intervention deeply rooted in the schools settings and immediately replicable, but also the disadvantages of necessarily accepting the requests present in schools: in this case the information and involvement of teachers and the adoption of an inclusion model where children in difficulty work together with children without problems. We hypothesized that a group training of executive functions (in particular impulse control, controlled attention, and working memory) carried out within the routine day activities of a kindergarten and interesting at the same time children with ADHD symptoms and TD children could be well accepted by children, school, and parents and could improve children’s executive functions, possibly also reducing ADHD symptoms.

## Materials and Methods

### Participants

#### Children Exhibiting ADHD Symptoms

The sample consisted of 26 children attending their last year of pre-school (kindergarten) and who exhibited ADHD symptoms but without a diagnosis, due to the fact the Italian guidelines on ADHD suggest to avoid the complete assessment and the diagnosis before six. Children were considered as exhibiting ADHD symptoms on the basis of information collected from teachers and a validated rating scale for teachers, the IPPDAI “Identificazione Precoce del Disturbo da Deficit di Attenzione/iperattività per Insegnanti” (“Early Identification of ADHD for Teachers,” Re and Cornoldi, 2009), and on the basis of information collected from parents through interviews and another rating scale whenever possible (IPDDAG, Re and Cornoldi, 2009).

The IPDDAI includes 14 items referring to symptoms described both by DSM-IV and DSM-5 (American Psychiatric Association, 1994, 2013) identified as the most predictive of ADHD in preschoolers, seven concerning inattention (items 1, 2, 4, 5, 12, 13, and 14), seven concerning hyperactivity/impulsivity (items 3, 6, 7, 8, 9, 10, and 11), and four additional items (15, 16, 17, and 18) concerning risk factors, i.e., the fact of “coming from a disadvantaged family” (item 15), “having problematic situations at home” (item 16), “having poor cognitive abilities” (item 17), and “having emotional and relational problems” (item 18). The IPDDAI scale has been validated and standardized for the Italian population. Test–retest information is only available for the version for older children (*r* = 0.80) but, in a study correlating the IPDDAI scores given by kindergarten teachers with the identification of ADHD symptoms 1 year later by primary school teachers, Marcotto et al. (2002) identified a positive correlation of *r* = 0.56. Moreover, IPDDAI scores appear to be highly correlated with the ADHD score obtained with the Conners’ scale for both inattention (*r* = 0.88) and hyperactivity (*r* = 0.84; Trevisi and Re, 2008). The IPDDAG scale has the same structure of IPDDAI but refers to home situation.

Teachers and parents indicated the presence of each behavior by using a 4-point Likert scale, ranging from 0 to 3 (0 = behavior never present/not at all, 1 = behavior sometimes present, 2 = behavior often present, 3 = behavior always/very much present). After combining the values of the seven ratings into two subscale scores, the children of the ADHD group had a score greater than 11 (corresponding to the 10° percentile) either in the attention or in the hyperactivity subscale of IPDDAI or both.

The children with ADHD symptoms were randomly assigned to two conditions as follows: 13 children (eight boys and five girls, mean age = 63.42, SD = 4.98) were included in the experimental training (hereafter referred to as the training condition) and 13 (nine boys and four girls, mean age = 63.03, SD = 4.40) in the non-training condition where children had business as usual or activities devoted to develop literacy. Children of both groups had similar scores on the IPPDAI rating scale: training group inattention, *M* = 11.23 (SD = 3.41); hyperactivity, *M* = 10.57 (SD = 5.35); control group inattention, *M* = 11.04 (SD = 4.83); and hyperactivity *M* = 11.8 (SD = 4.61).

#### Typically Developing Children

As schools required that children should be trained within an integration perspective that did not isolate the children with problems, children with ADHD symptoms were trained together with typically developing children (TD, with an IPPDAI score of below 3, i.e., >50° percentile). Therefore, we individuated 26 TD children; of these 13 (five boys and eight girls, mean age = 65.15, SD = 4.498) were randomly assigned to the training condition and 13 to the non-training condition (six boys and seven girls, mean age = 65.61, SD = 4.21). In the selection of the TD children, who had to be included in the groups, we had to decide whether to maintain the same proportions of males and females present in the ADHD symptoms groups (with a larger presence of boys) or to have more homogeneous groups by compensating the proportion of males and females, by including a larger number of girls. As the study design did not include comparisons between children with ADHD symptoms and TD children (but only between treated and untreated children), after a discussion with the teachers, we decided for the second alternative.

For all students involved in this investigation, we received appropriate approvals from their parents and schools. This study was carried out in accordance with the recommendations of “the ethic committee of the University of Padova.”

Based on the outcomes of IPDDAI and on interviews with the teachers, children with low socioeconomic status, poor intellectual abilities (as measured by the IPDDAI specific control items), family or other relevant problems, and finally children that belonged to foreign communities were excluded from the sample. All the students were Caucasian; had no physical, sensory, or neurological impairments; spoke Italian fluently; and had grown up in an adequate socio-cultural environment.

### Procedure

The procedure was defined on the basis of schools’ constraints. In particular, after the administration of the teachers’ rating scale and before the training, we were allowed to administer to all children a stop-signal test (Walk–No Walk Test [Ranette], Marzocchi et al., 2010). Furthermore, because we involved two different schools in the project, we were allowed to administer a second executive test, but this test was different in the two schools, according to their requests. In one school, a working memory test (the Dual Request Selective Task; Re and Cornoldi, 2007), and in the other one, an impulsivity control test was administered (Matching Figures MF-14; Marzocchi et al., 2010). The assessment was followed by 17 one-hour sessions distributed over a 9-week period, twice a week for the training group interested in executive functions and for the control group interested in the empowerment of cognitive functions, according to the usual school practices. One week after the end of the training, the teachers were invited to complete the IPDDAI again and the same measures collected before the trainings were recollected.

#### Walk–No Walk Test (Ranette; Frogs)

The Walk–No Walk Test ([Ranette], Marzocchi et al., 2010) is a paper-and-pencil test that evaluates control of attention and the inhibition of an ongoing response. It is derived from the “stop signal task” of Logan and Cowan (1984). The task requires children to follow a series of directions and stop an ongoing response when a particular event (a signal) occurs.

The test includes two A4 sheets of paper in which 20 stairs (one for each trial) are drawn with a little frog on the first step. The child is asked to cancel a step each time he or she hears the GO signal, while she/he has to stop every time she/he hears the STOP signal. The STOP signal is very similar to the GO signal but is different in its ending. Obviously, for every trial, there are many GO signals and only one STOP signal. The difficulty of this task is that the STOP signal is made in two parts, and the first part has the same sound as the GO signal. Therefore, the child must wait to hear the entire sound before providing the response in order to understand if it is a GO or STOP signal. The score is defined by the number of correct trials. Test–retest reliability of the test is *r* = 0.70.

#### Supplementary Assessment

##### The Working Memory Dual Request Selective Task (DRST)

The Dual Request Selective Task (DRST; Re and Cornoldi, 2007; see also Lanfranchi et al., 2004) is a visual spatial working memory task that assesses the ability to control information maintenance in working memory and to inhibit irrelevant information. The test is based on a 4 × 4 matrix (17 cm × 17 cm), divided into 16 cells. The matrix is blank with a red square always situated in the same position. DRST requires the children to perform a double task:

(1) Remember the first position indicated by the experimenter.
(2) Clapping hands when the experimenter indicates the red square.

To make the task more attractive, a small plastic frog is shown moving into the matrix.

There are 10 trials in order by difficulty level. Difficulty depends on the number of cells touched by the frog (length of the pathway) from a minimum of two to a maximum of six cells. There are two trials for each length level. The child must complete the entire task.

A trial is considered correct only when the child carries out both tasks correctly; in other words, clapping and remembering the first position. Also errors are considered in the task as they seem to represent a specific element of weakness in the case of children with ADHD symptoms (Cornoldi et al., 2001). Average time for this task is 10 min.

Cronbach alpha reliability for this test is high (0.84, according to Lanfranchi et al., 2015).

##### MF 14

The MF-14 test (Marzocchi et al., 2010) is derived from the impulsivity control MFFT test (Kagan, 1966). It assesses several executive components and, in particular, sustained attention and impulsivity control. The test consists of 14 items that include a target picture and six alternative pictures similar to the target. Among these pictures, only one is exactly like the target. The child has to identify the picture that is just like the target. The pictures represent everyday life objects. For the scoring of this test two parameters are considered:

• Number of errors.
• Response time (i.e., the time of the first response) that is assumed to represent a form of impulsivity.

Despite the fact that test–retest reliability collected in different studies and reported in the Manual (Marzocchi et al., 2010) is moderate both for errors (ranging between 0.49 and 0.60) and for response time (ranging between 0.41 and 0.50), the test has been validated and successfully used in a large number of studies (see Marzocchi et al., 2010).

### Training

The training consisted of 17 sessions, each lasting 1 h, administered twice a week to the whole group of children (with ADHD symptoms and TD) separately for each school. The training (for some examples, see Re and Cornoldi, 2007) used activities presented in the published manual *Sviluppare la concentrazione e l’autoregolazione* (*Development of Concentration and Self-Control;* Re and Cornoldi, 2007; Caponi et al., 2008, 2009a,b) and was carried out by trained psychologists one per school. The activities proposed to the children can be divided in four main blocks:

(1) *Block 1:* The first two units introduced the behavioral strategies to maintain control and stay on task. The focus of these units was on the correct behaviors favoring the maintenance of attention (such as the right posture, inhibition of impulsive movements, focalization of the vision), self-control (monitoring of comprehension and attention), the control of the impulsive response (“don’t give a hurried answer,” “think and wait your turn before answering”), and maintenance and control of information in working memory. A nursery rhyme and a dummy were presented at the beginning of every unit to indicate the beginning of the specific activity.
(2) *Block 2:* The following six units trained selective attention, selective working memory based on a criterion, and the capacity of inhibiting impulsive responses. Games requiring paper and a pencil or a motor activity were proposed.
(3) *Block 3:* The next six units are related to sustained attention and the ability of considering the whole stimulus before giving an answer. The objective of these units was to increase the time of sustained attention and to increase the awareness of the time necessary to do an activity. Moreover there were other activities on selective working memory in association with an interpolated task.
(4) *Block 4:* The final three units were dedicated to divided attention and shifted attention, or the ability to pay attention to two different stimuli simultaneously and to shift attention from a stimulus to another one, and on updating the information in working memory.

The training did not include activities directly related with the pre- and post-measures. Each session always had the same structure, as follows:

(1) *Metacognitive introduction:* The teacher captured the children’s attention and commented on the goal of the day’s activities.
(2) *Presentation of the cognitive requests:* The teacher explained the activities for the day.
(3) *Instructions and preliminary practice with the task of the day*.
(4) *Organization of task:* The teacher organized the activity and eventually divided the children in subgroups.
(5) *Practice with the complete task:* The teacher invited the children to do the complete task.
(6) *Promotion of strategic reflections:* The teacher asked the children to comment on the activities and report strategies that they had used or thought they could use. The teacher guided the children towards the indication of strategies.
(7) *Introspection and feedback.* The teacher asked to the children how well they thought they did the task, gave feedback to the children, and discussed reasons for eventual failures.

In the control condition, children were provided with an equivalent amount of time working on typical school activities, for example pre-reading and pre-writing exercises. These activities were carried out by the same psychologists who conducted the training.

### Fidelity of Implementation

In order to have high fidelity in the implementation, the training was carried out by psychologists specifically knowledgeable about the use of the present program and all the activities were available in written form. The authors of the present paper had supervision meetings with the trainers every 2 weeks. During the training, the trainer maintained a daily journal of activities undertaken in each session. In each case, observed activities highly corresponded to the intended components of the lessons: in fact in 90% of the cases the activities were rated as perfectly corresponding to the training Manual. A written record was also maintained and observations and supervision sessions were carried out for the control condition by considering the topics of each session.

## Results

The training was well received both by the children and the teachers who attended the sessions. Also parents expressed a positive impression of the project and some of them reported observations of the effective improvements for their children.

Concerning the data analysis, we compared trained vs. non-trained groups on the pre-test and, despite minor differences, did not find any significant differences between the trained and non-trained children with ADHD symptoms groups and between the trained and the non-trained TD groups, whereas the overall group of children with ADHD symptoms had a poorer performance than the overall group of TD children. As the experimental design was related to children with ADHD and the TD children were involved only in order to meet a school request and the selection of measures was calibrated on the characteristics of ADHD children, we decided to examine in the first instance the case of children with ADHD. Therefore we analyzed the data concerning children with ADHD symptoms using a group (training vs. non-training) by time (pre- vs. post-training) analysis of variance (ANOVA). As a further control, we examined whether the training had an effect on TD children. In addition, we analyzed the results with a clinical approach. Based upon the guidelines produced by the Italian National Consensus Conference (2007) on LD and associated recommendations (Tressoldi and Vio, 2008) and predefining a positive change of at least 1 SD to represent clinical improvement, we considered the percentage of participants who had such a positive change.

Considering the performance of children with ADHD symptoms on the Walk–No Walk Test (Ranette), we found a significant main effect of time *F*(1,24) = 17.67, *p* < 0.001, $\eta_{p}^{2}$ = 0.42. We did not find a significant main effect of groups (*F* < 1), but we found a significant interaction *F*(1,24) = 8.92, *p* < 0.006, $\eta_{p}^{2}$ = 0.27. *Post hoc* comparisons showed that the training group significantly improved (*p* < 0.001), whereas the slight increase in performance of the non-training group was far from significance (*p* = 0.40). A comparison between the two schools showed that the benefits of the training were similar in the two school systems [school A: ADHD symptoms training group pre *M* = 4.41 (SD = 3.64), post *M* = 9.86 (SD = 3.93); ADHD symptoms non-training group pre *M* = 6.57 (SD = 3.78), post *M* = 8.71 (SD = 5.09); school B: ADHD symptoms training group pre *M* = 8.67 (SD = 4.84), post *M* = 13.5 (SD = 2.81); ADHD symptoms non- training group pre *M* = 9.5 (SD = 6.92), post *M* = 8.83 (SD = 5.56)].

We found similar results with the supplementary tests. Indeed, for the errors at the *MF- 14 test* significant main effect of time *F*(1,12) = 8.33, *p* = 0.014, $\eta_{p}^{2}$ = 0.41 and interaction *F*(1,12) = 7.11, *p* = 0.021, $\eta_{p}^{2}$ = 0.37 were found, while we did not find a main effect of group (*F* < 1). Again, the interaction was due to the fact that children who followed the training improved their performance (*p* = 0.002), while the non-training group did not (*p* = 0.88). Concerning the *MF-14* response time, the difference between the pre- and post-measures, despite the fact that only approached the significance level, *F*(1,12) = 4.69, *p* = 0.051, was characterized by a substantial effect size, $\eta_{p}^{2}$ = 0.281. We did not find a main effect of group (*F* < 1) nor a significant interaction [*F*(1,12) = 3.49, *p* = 0.086, $\eta_{p}^{2}$ = 0.225], even if the mean scores showed that only the trained group became slower, i.e., more reflective in responding (pre-training *M* = 9.58, SD = 6.35; post-training *M* = 18.07, SD = 15.35), while the other group did not change (pre-training *M* = 10.09, SD = 3.32; post-training *M* = 10.71, SD = 5.60).

Considering the correct responses at the DRST task, we found a significant main effect of time *F*(1,10) = 10.92, *p* = 0.008, $\eta_{p}^{2}$ = 0.52, but we did not find a significant main effect of group or a significant interaction. For errors of DRST, we found the same pattern of results, i.e., a significant main effect of time *F*(1,10) = 15.21, *p* = 0.003, $\eta_{p}^{2}$ = 0.60, but no other significant effects. However, mean scores showed that the reduction of errors after the training was wider in the case of training group (pre-training *M* = 9.17, SD = 4.92; post-training *M* = 4.83, SD = 3.31) than in the other group (pre-training *M* = 6.67, SD = 3.61; post-training *M* = 4, SD = 2.83).

Finally, we analyzed the ratings given to the children on the IPDDAI rating scale by their teachers, and we found a main effect of time for the inattention subscale *F*(1,24) = 28.86, *p* < 0.001, $\eta_{p}^{2}$ = 0.55, while we did not find a significant group effect or interaction. We found the same pattern of results for the hyperactive subscale, i.e., only a main effect of time *F*(1,24) = 33.61, *p* < 0.001, $\eta_{p}^{2}$ = 0.58. In this case, as it can be seen in **Table 1**, according to the teachers, both groups (training and non-training) improved their behavior, and this happened to the same extent.

**Table 1:** Mean scores obtained by the two groups (training and non-training) of children with Attention Deficit Hyperactivity Disorder (ADHD) symptoms before and after the training.

		Training		Non-training	
	*N*	*M*	SD	*M*	SD
**Teacher rating scale**					
IPDDAI Inattention pre	13	11.23	3.41	11.04	4.83
IPDDAI Inattention post	13	8.5	2.55	7.8	3.63
IPDDAI hyperactivity pre	13	10.57	5.35	11.8	4.61
IPDDAI hyperactivity post	13	6.92	4.32	8	4.10
**Executive Function Tests**					
Walk–Nowalk pre Correct trials	13	6.54	4.54	7.92	5.42
Walk–Nowalk Ranette post Correct trials	13	11.54	3.82	8.77	5.08
MF errors pre	7	25.71	10.09	20.57	6.29
MF errors post	7	14.86	7.56	20.14	9.51
MF time pre	7	9.58	6.35	10.09	3.32
MF time post	7	18.07	15.35	10.71	5.60
DRST correct responses pre	6	3.00	3.03	4.33	2.80
DRST correct responses post	6	5.67	2.66	6.50	2.07
DRST err pre	6	9.17	4.92	6.67	3.61
DRST err post	6	4.83	3.31	4.00	2.83

The fact that we were required to include typically developing children in the trained groups offered the possibility of examining whether the training affected them, despite the fact it had been designed for children with ADHD symptoms. In fact, for the test administered to all the children, i.e., the Walk–No Walk Test (Ranette), we found a significant effect of time *F*(1,24) = 10.51, *p* = 0.003, $\eta_{p}^{2}$ = 0.305, but we did not find neither the effect of group [*F*(1,24) = 1.97, *p* > 0.05, $\eta_{p}^{2}$ = 0.076] nor the interaction (*F* < 1). However, mean scores showed some improvement for the trained group and not for the control one (see **Table 2**). Concerning the other two supplementary tests, we only found, for the correct responses on the DRST, a main effect of time [*F*(1,10) = 6.10, *p* = 0.033, $\eta_{p}^{2}$ = 0.379]. On the contrary we did not find significant differences between groups, but the improvements were always more evident in the trained group. In the case of errors in the DRST task, only the group that followed the training reduced their number of errors (see **Table 2**). Concerning teachers’ ratings of TD with the IPDDAI, there were no clear trends because the scores were already very low before the training.

**Table 2:** Mean scores obtained by the two groups (training and non-training) of children with Typical Development.

		Training		Non-training	
	*N*	*M*	SD	*M*	SD
**Teacher rating scale**					
IPDDAI Inattention pre	13	3.35	2.44	3.57	1.89
IPDDAI Inattention post	13	3.34	2.21	2.15	1.30
IPDDAI hyperactivity pre	13	2.84	2.04	3.69	2.28
IPDDAI hyperactivity post	13	2.19	1.92	2.96	2.62
**Executive Function Tests**					
Walk–Nowalk pre Correct trials	13	12.08	3.90	11	4.06
Walk–Nowalk Ranette post Correct trials	13	15.39	2.96	13.23	3.59
MF errors pre	7	17.57	10.89	18	7.96
MF errors post	7	12.57	10.55	15.86	5.15
MF time pre	7	12.89	8.55	20.47	26.99
MF time post	7	23.50	18.85	13.59	8.65
DRST correct responses pre	6	3.67	2.66	5.67	30.1
DRST correct responses post	6	6.33	0.82	5.83	1.72
DRST err pre	6	8	4.10	5.67	4.46
DRST err post	6	4	1.26	4.83	2.32

### Clinical Change

**Table 3** displays the effect sizes of the changes and the number of participants meeting the clinical criteria (of an improvement of at least 1 SD). This type of analysis reveals specific improvements that may be negligible when group averages are analyzed, but it may be very important for the individual student.

**Table 3:** Clinical Comparison: number and frequencies of children of the ADHD group who changed of at least 1 SD from the pre to the post training, in the Training and Non-Training condition.

	ADHD		
Task	Training	Non-Training	*d*
IPDDAI Inattention	6/13 (46.15%)	4/13 (30.77%)	0.41
IPDDAI Hyperactivity	3/13 (23.08%)	4/13 (30.77%)	–0.23
Walk–Nowalk correct trials	6/13 (46.15%)	1/13 (7.69%)	1.33
MF errors	4/7 (57.14%)	0/7	2.37
MF time	1/7 (14.28%)	0/7	1.12
DRST correct responses	2/6 (33.33%)	1/6 (16.66%)	0.54
DRST errors	2/6 (33.33%)	1/6 (16.66%)	0.54

Based on the clinical significance criteria, the training clearly improved students’ performance compared with the non-trained children in all parameters, except for hyperactivity of the IPPDAI rating scale, with an effect size ranging from 0.41 (for IPDDAI inattention) to 2.37 (Walk–No Walk Test [Ranette]). Cohen’s *d* was calculated from the log odds as follows (Borenstein, 2009): *d = (ln(o)sqrt(3))/(pi).*

## Conclusion

The main purpose of this work was to promote the executive functions of children with ADHD symptoms in the unique and delicate period represented by their preschool years. For this purpose, we conducted training on controlled attention, control of impulsive response, and working memory with 5-year-old children. The training had a metacognitive approach and aimed to improve the children’s attention capacity, the control of their behavior, and information in working memory through playful activities that required them to maintain attention or to control their behavior. Children with ADHD symptoms were randomly assigned to the training condition and to the control condition involving school activities. As the schools required that the children were trained with other children who have a typical development in an integration perspective that did not isolate the children with ADHD symptoms, the typically developing children indicated by the two schools were trained together with the children exhibiting ADHD symptoms.

Results suggest that a training of executive functions may be effective although its effect was more evident in some measures (a significant interaction between training and phases was observed only for Walk–No Walk Test [Ranette] and the errors in MF-14), than in others. Moreover, the children with a TD who took part in the training improved their competences as well. The effects, however, were less evident for the associated inattentive and hyperactive problems as rated by the teachers, as the trained group actually improved, but a similar improvement was observed in the control group. Therefore, part of the improvement seemed to be due to the general activities proposed during this period to the children and to their associated maturation. In fact it should be noticed that, despite the fact that the period between the two compilations of the IPDDAI scale by teachers was relatively short (around four months) ratings significantly changed.

It must be noticed that the project required that the teachers rating the children shared the goals and the method and were therefore informed about the formation of the groups. This is a strength of the method but also a weakness for the interpretation of the teachers’ ratings. However this bias did not seem to produce an optimistic view of the reduction of the symptoms in the training group. Actually, the bias could also have been in the opposite direction, bringing the teachers to pay more attention to the symptoms presented by the treated children.

Finally, based on the clinical significance criterion, that considered an improvement of at least one SD as a significant clinical change, we saw that the training group improved the performance of children with ADHD symptoms by comparison with the corresponding children of the non-training condition, further supporting the hypothesis that an intervention for controlled attention, control of impulsive behavior and working memory is possible at an early age. As executive functions are related with a series of school activities (e.g., comprehension, expressive writing, problem solving, etc.), we can hypothesize that the benefits can be extended to various aspects of schooling. However, in this study, we were not allowed to assess for far transfer effects, and the only general measure we had, based on the teachers’ perceptions of attention and hyperactivity problems, did not reveal a training benefit. Only future research will be able to better understand this point.

Nevertheless, important clinical implications can be derived from this research. First, we have new evidence of the possibility of administering the training of executive functions to preschool children who exhibit ADHD symptoms. The present cognitive training had the advantages of being easily implemented within the preschoolers’ usual activities; well received by children, teachers, and parents; and produced specific effects related to the structure and the pre-established goals of the program. However, it seems important to try to prevent subsequent severe consequences for primary school children, not only at the level of cognitive functioning but also at the level of the typically associated problems. Indeed, in kindergarten, children are more flexible, relations with peers, and with parents are still easily modifiable, and negative experiences can be avoided (Sonuga-Barke et al., 2006). Working with very young children can also help to prevent negative consequences on self-esteem and motivation and, as suggested by Kern et al. (2007), reduce the appearance of oppositional or deviant behaviors. Our study tried to offer a contribution in this direction, but it is in need of replication and generalization. In fact, our study presents a series of limitations including the small number of children trained, the small number and the modest reliability of measures we were allowed to use and the specificity of the observed effects, the impossibility to have individual clinical profiles of the children and to examine the factors that could explain why some children improved and others did not, and the modest involvement of their parents.

Even considering these limitations, our findings show that great attention should be devoted to early cognitive interventions for children with ADHD or exhibiting ADHD symptoms. Indeed, even if a diagnosis of ADHD is difficult in the preschool years, early identification and intervention could be very beneficial for the future of these children.

## Conflict of Interest Statement

The Editor Silvia Lanfranchi declares that, despite being affiliated to the same institution as the authors Anna M. Re, Agnese Capodieci and Cesare Cornoldi, the review process was handled objectively and no conflict of interest exists. The authors declare that the research was conducted in the absence of any commercial or financial relationships that could be construed as a potential conflict of interest.

## References

[B1] American Psychiatric Association. (1994). *Diagnostic and Statistical Manual of Mental Disorders* 4th Edn (DSM - IV). Washington DC: American Psychiatric Association.

[B2] American Psychiatric Association. (2013). *Diagnostic and Statistical Manual of Mental Disorders* 5th Edn. Washington, DC: American Psychiatric Association.

[B3] Bergman Nutley S. Söderqvist S. Bryde S. Thorell L. B. Humphreys K. Klingberg T. (2011). Gains in fluid intelligence after training non-verbal reasoning in 4-year-old children: a controlled, randomized study. *Dev. Sci.* 14 591–601. 10.1111/j.1467-7687.2010.01022.x 21477197

[B4] Borenstein M. (2009). “Effect sizes for continuous data,” in *The Handbook of Research Synthesis and Meta Analysis* eds Cooper H. Hedges L. V. Valentine J. C. (New York, NY: Russell Sage Foundation) 279–293.

[B5] Caponi B. Clama L. Re A. M. Cornoldi C. (2008). *Sviluppare la Concentrazione e L*’*autoregolazione - Vol. 1 Giochi e Attività Sul Controllo Attentivo*. [*Developingt Concentration and Self-Control- Vol-1 Activities on Attention Control*]. Trento: Erickson.

[B6] Caponi B. Clama L. Re A. M. Cornoldi C. (2009a). *Sviluppare la Concentrazione e L*’*autoregolazione - Vol. 2 Giochi e Attività Sul Controllo Della Risposta Impulsiva*. [*Developing Concentration and Self-Control- Vol-2 Activities on Control of Impulsive Response*]. Trento: Erickson.

[B7] Caponi B. Clama L. Re A. M. Cornoldi C. (2009b). *Sviluppare La Concentrazione e L*’*autoregolazione: Giochi e Attività Sul Controllo Della Memoria di Lavoro*, Vol. 3. [*Developing Concentration and Self-Control- Vol-3 Activities on Control of Working Memory*]. Trento: Erickson.

[B8] Consensus Conference. (2007). Consensus Conference Sui Disturbi Evolutivi Specifici Dell’Apprendimento [Consensus Conference on Learning Disabilities]. Milano: Circolo della Stampa.

[B9] Cornoldi C. Marzocchi G. M. Belotti M. Caroli M. G. Meo T. Braga C. (2001). Working memory interference control deficit in children referred by teachers for ADHD symptoms. *Child Neuropsychol.* 7 230–240. 10.1076/chin.7.4.230.8735 16210212

[B10] Diamond A. (2012). Activities and programs that improve children’s executive functions. *Curr. Dir. Psychol. Sci.* 21 335–341. 10.1177/0963721412453722 25328287 PMC4200392

[B11] DuPaul G. J. Kern L. (2011). *Young Children With ADHD: Early Identification and Intervention.* Washington DC: American Psychiatric Association 10.1037/12311-000

[B12] Evans S. W. Owens J. S. Bunford N. (2013). Evidence-based psychosocial treatments for children and adolescents with attention-deficit/hyperactivity disorder. *J. Clin. Child Adolesc. Psychol.* 43 527–551. 10.1080/15374416.2013.850700 24245813 PMC4025987

[B13] Halperin J. M. Marks D. J. Bedard A. C. V. Chacko A. Curchack J. T. Yoon C. A. (2013). Training executive, attention, and motor skills: a proof-of-concept study in preschool children with ADHD. *J. Atten. Disord.* 17 711–721. 10.1177/1087054711435681 22392551

[B14] Kagan J. (1966). Reflection-impulsivity: the generality and dynamics of conceptual tempo. *J. Abnorm. Psychol.* 71 17–24. 10.1037/h0022886 5902550

[B15] Kern L. DuPaul G. J. Volpe R. Sokol N. Lutz J. G. Arbolino L. (2007). Multi-setting assessment-based intervention for young children at-risk for ADHD: initial effects on academic and behavioral functioning. *Sch. Psychol. Rev.* 36 237–255.

[B16] Lanfranchi S. Cornoldi C. Vianello R. (2004). Verbal and visuo-spatial working memory deficit in children with Down Syndrome. *Am. J. Ment. Retard.* 109 456–466. 10.1352/0895-8017 15471512

[B17] Lanfranchi S. De Mori L. Mammarella I. C. Carretti B. Vianello R. (2015). Spatial-sequential and spatial-simultaneous working memory in individuals with Williams Syndrome. *Am. J. Intellect. Dev. Disabil.* 120 193–202. 10.1352/1944-7558-120.3.193 25928432

[B18] Logan G. D. Cowan W. B. (1984). On the ability to inhibit thought and action: a theory of an act of control. *Psychol. Rev.* 91 295–327. 10.1037/0033-295X.91.3.295

[B19] Marcotto E. Paltenghi B. Cornoldi C. (2002). La scala IPDDAI: contributo per la costruzione di uno strumento per l’identificazione precoce del DDAI. [The IPDDAI Scale: a contribution for the construction of a scale for the early identification of ADHD]. *Difficoltà di Apprendimento* 8 153–172.

[B20] Mariani M. A. Barkley R. A. (1997). Neuropsychological and academic functioning in preschool boys with attention deficit hyperactivity disorder. *Dev. Neuropsychol.* 13 111–129. 10.1080/87565649709540671

[B21] Martinussen R. Hayden J. Hogg-Johnson S. Tannock R. (2005). A meta-analysis of working memory impairments in children with attention-deficit/hyperactivity disorder. *J. Am. Acad. Child Adolesc. Psychiatry* 44 377–384. 10.1097/01.chi.0000153228.72591.73 15782085

[B22] Marzocchi G. M. Re A. M. Cornoldi C. (2010). *BIA – Batteria Italiana per l*’*ADHD* Trento: Erickson.

[B23] Miyake A. Friedman N. P. (2012). The nature and organization of individual differences in executive functions four general conclusions. *Curr. Dir. Psychol. Sci.* 21 8–14. 10.1177/0963721411429458 22773897 PMC3388901

[B24] Rajwan E. Chacko A. Moeller M. (2012). Nonpharmacological interventions for preschool ADHD: state of the evidence and implications for practice. *Prof. Psychol. Res. Pr.* 43 520–526. 10.1037/a0028812

[B25] Rapport M. D. Orban S. A. Kofler M. J. Friedman L. M. (2013). Do programs designed to train working memory, other executive functions, and attention benefit children with ADHD? A meta-analytic review of cognitive, academic, and behavioral outcomes. *Clin. Psychol. Rev.* 33 1237–1252. 10.1016/j.cpr.2013.08.005 24120258

[B26] Re A. M. Cornoldi C. (2007). ADHD at five: a diagnosis-intervention program. *Adv. Learn. Behav. Disabil.* 20 223–240. 10.1016/S0735-004X(07)20009-6

[B27] Re A. M. Cornoldi C. (2009). Two new rating scales for assessment of ADHD symptoms in Italian preschool children. *J. Atten. Disord.* 12 532–539. 10.1177/1087054708323001 18725657

[B28] Re A. M. De Franchis V. Cornoldi C. (2010). A working memory control deficit in kindergarten ADHD children. *Child Neuropsychol.* 16 134–144. 10.1080/09297004303373404 20104377

[B29] Reid R. Trout A. L. Schartz M. (2005). Self-Regulation interventions for children with attention deficit/hyperactivity disorder. *Except. Children* 71 361–377.

[B30] Röthlisberger M. Neuenschwander R. Cimeli P. Michel E. Roebers C. M. (2012). Improving executive functions in 5-and 6-year-olds: evaluation of a small group intervention in prekindergarten and kindergarten children. *Infant Child Dev.* 21 411–429. 10.1002/icd.752

[B31] Salvaguardia F. Re A. M. Caponi B. Cornoldi C. (2009). Esperienza di un training sulla memoria di lavoro con bambini con tratti di disattenzione e iperattività (Experience with a working memory training with children with disattention/hyperactivity traits). *Disturbi di attenzione iperattività* 4 171–185.

[B32] Schoemaker K. Bunte T. Wiebe S. A. Espy K. A. Dekovic M. Matthys W. (2012). Executive function deficits in preschool children with ADHD and DBD. *J. Child Psychol. Psychiatry* 53 111–119. 10.1111/j.1469-7610.2011.02468.x 22022931

[B33] Sinzig J. Vinzelberg I. Evers D. Lehmkuhl G. (2014). Executive function and attention profiles in preschool and elementary school children with autism spectrum disorders or ADHD. *Int. J. Dev. Disabil.* 60 144–154. 10.1179/2047387714Y.0000000040

[B34] Sonuga-Barke E. J. S. Dalen L. Daley D. Remington B. (2002). Are planning, working memory, and inhibition associated with individual differences in preschool ADHD symptoms? *Dev. Neuropsychol*. 21 255–272. 10.1207/S15326942DN2103_3 12233938

[B35] Sonuga-Barke E. J. S. Daley D. Thompson M. Laver-Bradbury C. Weeks A. (2001). Parent-based therapies for preschool attention-deficit/hyperactivity disorders: a randomized, controlled trial with a community sample. *J. Am. Acad. Child Adolesc. Psychiatry* 40 402–408. 10.1097/00004583-200104000-00008 11314565

[B36] Sonuga-Barke E. J. S. Thompson M. Abikoff H. Klein R. Brotman L. M. (2006). Nonpharmacological interventions for preschoolers with ADHD: the case for specialized parent training. *Infants Young Children* 19 142–153. 10.1097/00001163-200604000-00007

[B37] Thorell L. B. Lindqvist S. Bergman Nutley S. Bohlin G. Klingberg T. (2009). Training and transfer effects of executive functions in preschool children. *Dev. Sci.* 12 106–113. 10.1111/j.1467-7687.2008.00745.x 19120418

[B38] Tressoldi P. E. Vio C. (2008). Significatività clinica negli studi di efficacia dei trattamenti per i disturbi dell’apprendimento: una proposta. (Clinical significance in the studies on the efficacy of treatments of learning disorders: a proposal). *Psicol. Clin. dello Sviluppo XII* 2 291–301.

[B39] Trevisi G. Re A. M. (2008). Profili associati al disturbo da deficit di attenzione e iperattività nella scuola dell’infanzia. *Difficoltà di Attenzione e Iperattività* 4 9–23.

[B40] Willcutt E. G. Doyle A. E. Nigg J. T. Stephen V. Faraone S. V. Pennington B. F. (2005). Validity of the executive function theory of attention-deficit/hyperactivity disorder: a meta-analytic review. *Biol. Psychiatry* 57 1336–1346. 10.1016/j.biopsych.2005.02.006 15950006

[B41] Young S. Amarasinghe J. M. (2010). Practitioner review: non-pharmacological treatments for ADHD: a lifespan approach. *J. Child Psychol. Psychiatry* 51 116–133. 10.1111/j.1469-7610.2009.02191.x 19891745

